# Genomic and phenotypic insights into the novel species *Selenomonas lamontii* type strain ATCC 33150, currently described as *Selenomonas sputigena*

**DOI:** 10.1128/spectrum.00341-26

**Published:** 2026-06-16

**Authors:** Colin G. Hawkes, Blake O. Carroll, Aidan D. Moylan, Margaret E. J. Stiker, Taojun Wang, Myrna G. Serrano, Jason M. Ridlon, Daniel P. Miller

**Affiliations:** 1 School of Dentistry, Philips Institute for Oral Health Research, Virginia Commonwealth University 224030, Richmond, Virginia, USA; 2 Department of Microbiology and Immunology, School of Medicine, Virginia Commonwealth University 6889, Richmond, Virginia, USA; 3 Department of Animal Sciences, University of Illinois Urbana-Champaign 14589, Urbana, Illinois, USA; 4 Carl R. Woese Institute for Genomic Biology 124518, Urbana, Illinois, USA; 5 Division of Nutritional Sciences, University of Illinois at Urbana-Champaign 14589, Urbana, Illinois, USA; 6 Cancer Center at Illinois, University of Illinois at Urbana-Champaign 14589, Urbana, Illinois, USA; 7 Center for Advanced Study, University of Illinois Urbana-Champaign 14589, Urbana, Illinois, USA; The Ohio State University College of Dentistry, Columbus, Ohio, USA

**Keywords:** periodontitis, gingivitis, *Selenomonas*, periodontal disease

## Abstract

*Selenomonas sputigena* is an anaerobic, gram-negative bacterium found in the human mouth and upper respiratory tract. This organism is emerging as an important contributor to human health and disease. In the oral cavity, *S. sputigena* contributes to periodontitis and is associated with early childhood caries. Much of our current understanding of the genus *Selenomonas* and its relation to human health derives from studies of a single species, *S. sputigena*, and is further limited to the type strain, ATCC 35185. As *S. sputigena* is emerging as a significant contributor to human health, we sought to characterize the *S. sputigena* ATCC 33150 strain. Genomic analyses revealed that ATCC 33150, previously described as *S. sputigena*, is a novel *Selenomonas* sp., and we propose the name *Selenomonas lamontii*. Phenotypic comparison to *S. sputigena* reveals that *S. lamontii* grows more slowly and to a lower density *in vitro. S. lamontii* is more motile than *S. sputigena* and does not form surface-attached biofilms. Re-analysis of existing metagenomic data revealed the consistent presence of ATCC 33150 across all samples, with significantly elevated relative abundance in periodontitis-associated saliva compared to healthy donor controls. Collectively, we have identified ATCC 33150 as a new *Selenomonas* sp. and conducted one of the first direct comparative studies of traits relevant to colonization and persistence among *Selenomonas* spp.

**IMPORTANCE:**

Recognizing that strain ATCC 33150, historically described as *Selenomonas sputigena*, is a previously undescribed species has important implications for microbial systematics, physiology, and pathogenesis. Accurate taxonomic assignment underpins all downstream biological interpretation (e.g., comparative genomics, microbiome composition studies, virulence studies, and metabolic modeling). The identification of a novel species, therefore, refines the phylogenetic framework of the genus *Selenomonas*, enables more precise genotype-phenotype correlations, and may uncover previously unrecognized adaptations relevant to oral biofilm ecology and host interactions. Beyond taxonomy, this discovery strengthens the foundation and rigor of future mechanistic studies and provides context for discrepancies in previous studies involving this strain and ATCC 35185.

## INTRODUCTION

*Selenomonas* spp. are gram-negative, anaerobic, crescent-shaped bacilli with a distinctive flagellar bundle located on the concave surface of the cell (1). The genus *Selenomonas* comprises 16 cultivable and named species. *Selenomonas sputigena*, *Selenomonas noxia*, *Selenomonas flueggei*, *Selenomonas artemidis*, *Selenomonas infelix*, *Selenomonas dianae*, *Selenomonas felix* (2, 3), *Selenomonas timonae* (4), and *Selenomonas massiliensis* (5, 6) typically reside in the human mouth and upper respiratory tract (7). *Selenomonas ruminantium*, *Selenomonas caprae* (8), *Selenomonas ruminis* (9), *Selenomonas bovis* (10), and *Selenomonas montiformis* were isolated from the rumen of herbivores such as cattle, sheep, goats, and yaks. Two species are not host-associated. *Selenomonas lacticifex* is a beer spoilage organism, and *Selenomonas lipolytica* was isolated from a polluted tropical lagoon (11, 12). The genus *Selenomonas* belongs to the phylum Bacillota (formerly Firmicutes), class Negativicutes, order Selenomonadales, and family Veillonellaceae (1). Within Bacillota, typically characterized by bacteria with a single membrane (monoderm) and low G + C content, the Negativicutes form a distinct monophyletic clade comprising anaerobic bacteria with two membranes (diderms), such as *Veillonella*, *Megasphaera*, *Mitsoukella*, and *Dialister* (13). Interestingly, phylogenetic and evolutionary analyses based on conserved genetic markers suggest that Negativicutes display a combination of monoderm and diderm features and may represent an ancestral lineage within the Bacillota, in which the emergence of monoderms in the phylum resulted from the loss of the outer membrane (14).

Selenomonads may be among the earliest bacteria observed, as “animalcules” with morphology similar to theirs were described by van Leeuwenhoek from dental plaque in 1683 (15). During the 19th and 20th centuries, crescent-shaped, motile microorganisms were variously classified as protozoa or bacteria and were subsequently placed in a variety of genera (briefly reviewed by Chalcroft et al. [16]). Contradictory microscopic reports of the organisms’ flagella and cell wall structures led to disagreement over taxonomic classification (17, 18). Organisms later recognized as *Selenomonas* were initially classified within the genus *Spirillum,* likely because the individual bacteria can form spiral-like chains. In 1922, *Spirillum sputigenum* was formally reclassified as *Selenomonas sputigena* (Flugge 1886) by Boskamp (19). Although a type strain was eventually designated (ATCC 33150), subsequent work demonstrated that this strain was genetically and phenotypically divergent from other *S. sputigena* isolates, leading to its replacement with ATCC 35185 as the new type strain (20, 21).

*Selenomonas sputigena* has emerged as a clinically significant organism, being abundant in plaque biofilms from patients with severe periodontal disease (22–25). It has also been associated with deadly cases of septicemia (26–28). However, in the upper respiratory tract, *S. sputigena* may be protective against severe asthma by modulating the inflammatory response (29). Periodontal disease, or periodontitis, is a chronic inflammatory disease affecting the hard and soft tissues surrounding and supporting the teeth (30). Our recent work has demonstrated that *S. sputigena* adheres to and is internalized by gingival keratinocytes, thereby promoting a robust and sustained pro-inflammatory response, a hallmark of periodontitis (31, 32). In addition to periodontitis, *S. sputigena* exacerbates cariogenesis from *Streptococcus mutans*, promoting dental cavities (33). Its involvement in both subgingival and supragingival pathologies is unusual among oral bacteria and highlights the need for accurate strain-level taxonomy to guide functional studies.

In the study described here, we employ modern approaches to build upon the work of Johnson et al. (21) to further investigate the divergence of ATCC 33150 from *S. sputigena*. Recently, our group sequenced the whole genome of ATCC 33150 (34). Here, we employed whole-genome sequence analysis to compare the genome of ATCC 33150 with the genomes of 11 oral-isolate *Selenomonas* spp. and *S. ruminantium*. The resulting analyses reveal that ATCC 33150 is divergent from other oral selenomonads and therefore comprises a novel species within the genus. Here, we propose the new name for this organism, *Selenomonas lamontii*, in honor of Richard J. Lamont, an accomplished oral microbiologist. We next compared the genotypic and phenotypic characteristics of ATCC 33150 with those of *S. sputigena* type strain ATCC 35185. Genomic analyses revealed clusters of orthologous genes (COGs) unique to either ATCC 33150 or ATCC 35185. ATCC 33150 appears to have a more robust array of carbohydrate utilization pathways, whereas its genetic potential to scavenge iron is more limited than that of ATCC 35185. We then performed phenotypic comparison and revealed that ATCC 33150 grows more slowly in complex, nutrient-rich media. Compared to *S. sputigena* ATCC 35185*,* ATCC 33150 is highly aggregative during *in vitro* growth but does not form biofilms. We also determined that ATCC 33150 is more motile than *S. sputigena*. Taken together, our data indicate that ATCC 33150 is genetically and phenotypically divergent from *S. sputigena*. By reanalyzing previously collected metagenomic data sets, ATCC 33150 was identified in all samples and was positively associated with periodontitis, implicating it as a candidate contributor to periodontal disease.

A rigorous examination of microbes in association with human health requires results to be reproducible across different strains of a bacterial species. These findings advance our understanding of *Selenomonas* physiology, compare phenotypes associated with both periodontitis and caries, and provide valuable insights for future studies that will enhance the molecular toolbox for studying *Selenomonas* spp. This will greatly facilitate future studies to assess virulence factors critical to the molecular pathogenesis of oral disease and identify them as potential targets for therapeutic or vaccine development.

## RESULTS

### ATCC 33150 represents a new species of *Selenomonas*

**Fig 1:**
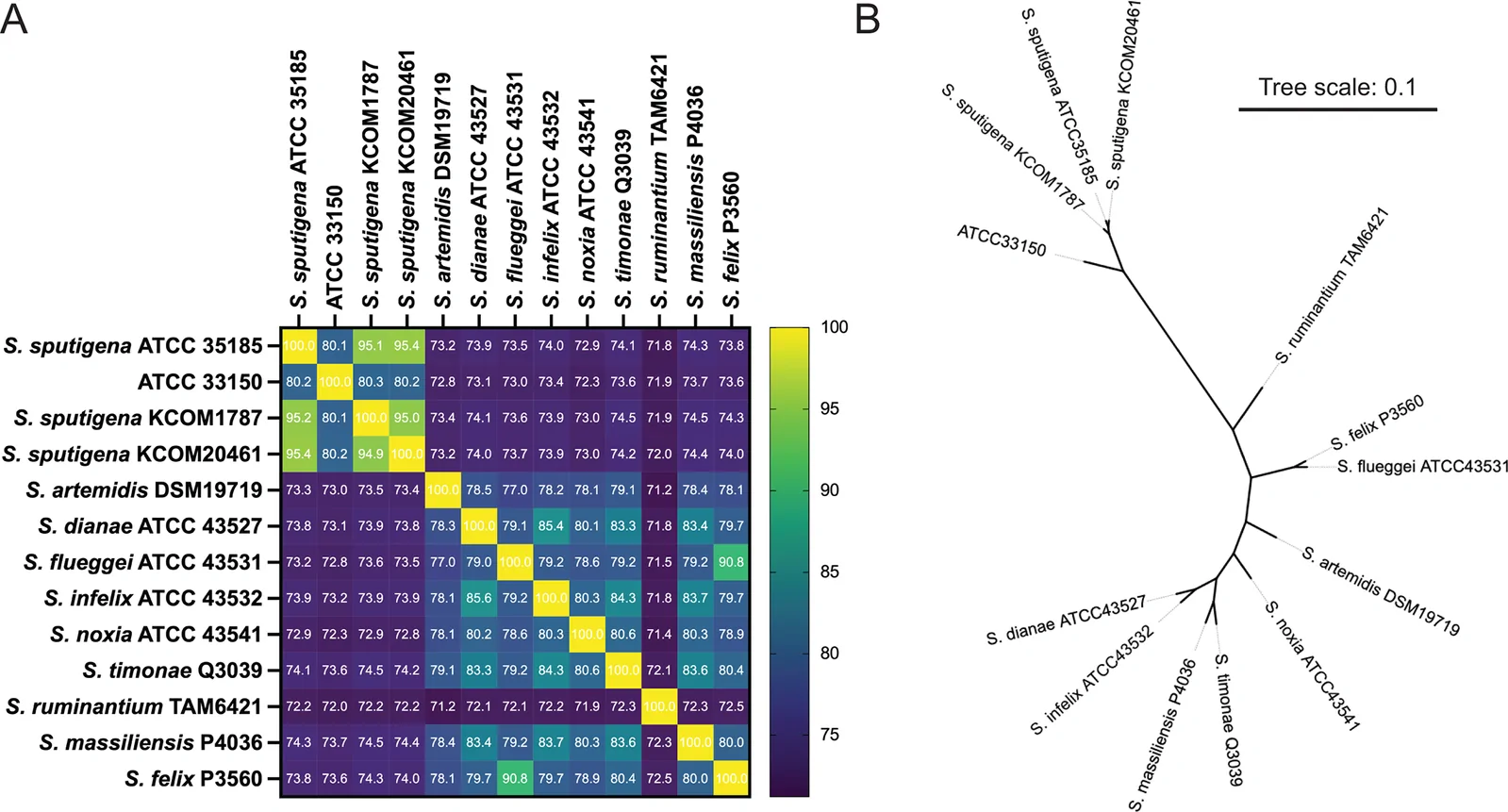
Comparative genomics suggests ATCC 33150 is a new species of *Selenomonas* that is most like *S. sputigena.* (**A**) The average nucleotide identity was determined for the named *Selenomonas* spp., and ATCC 33150 shares only 80.2% ANI with *S. sputigena* strains, which all share at least 95% ANI within the species, suggesting ATCC 33150 does not belong to *S. sputigena* but is a new *Selenomonas* species. (**B**) The phylogenetic tree demonstrates that ATCC 33150 is most evolutionarily related to *S. sputigena*.

Prior comparisons of the two ATCC strains described as *S. sputigena*, ATCC 33150 and ATCC 35185, revealed only 22% DNA homology using a DNA protection approach from S1 nuclease (21). We recently sequenced the genome of ATCC 33150 (34), and here, we compared the average nucleotide identity (ANI) of the ATCC 33150 genome with that of other *S. sputigena* genomes (ATCC 35185, KCOM1787, and KCOM20461) (Fig. 1A). We observed that ATCC 33150 shared only 80% ANI with each strain, which is significantly below the 95% threshold for taxonomic speciation, while the other strains shared 95% ANI. This provides strong scientific support for the previous supposition by the Judicial Commission of the International Committee of Systematic Bacteriology that the ATCC 33150 strain should not be classified as *Selenomonas sputigena* (20). We then sought to compare ATCC 33150 to genomes from other *Selenomonas* spp. (Fig. 1A). We found that it shared only 72%–74% ANI with oral selenomonads (*S. artemidis, S. flueggei, S. infelix, S. noxia, S. timonae,* and *S. dianae*) and 72% ANI with *S. ruminantium*, which resides in the rumen of sheep and cattle. The resulting phylogenetic tree places ATCC 33150 on a clear branch but within a clade with *S. sputigena*, distinct from the other oral *Selenomonas* spp. and *S. ruminantium* (Fig. 1B). These analyses demonstrate that ATCC 33150 represents a distinct species, and we propose that ATCC 33150 be reclassified as the type strain of *Selenomonas lamontii*.

### ATCC 33150 is a ubiquitous constituent of the oral microbiome and may be more abundant during periodontitis

**Fig 2:**
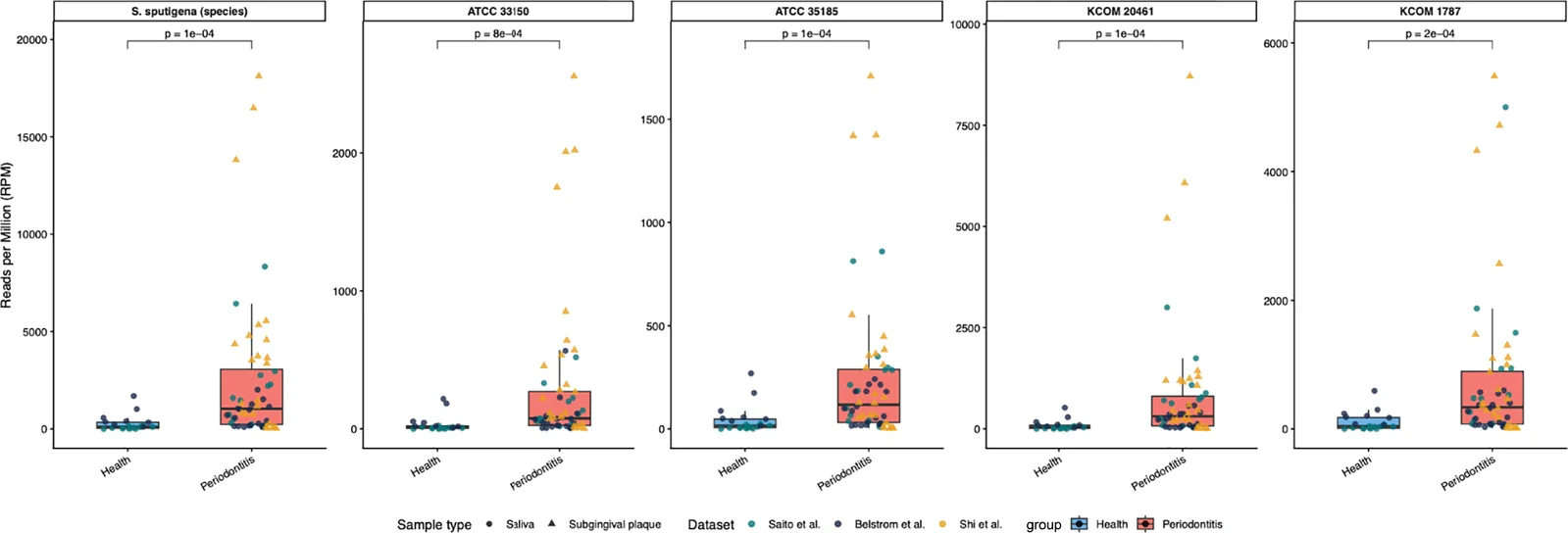
Abundance of the *Selenomonas* isolates in the linear mixed model combined metagenomic data set. The total abundance of the four strains currently annotated as *S. sputigena* was determined along with the abundance of each strain using a custom Kraken2 mini-database capable of detecting ATCC 33150, ATCC 35185, KCOM 20461, and KCOM 1787 at the subspecies level. All read counts were normalized to reads per million (RPM), models were fitted using the lme4 package, and *P*-values for fixed effects were obtained using the lmerTest package with Satterthwaite’s approximation for degrees of freedom.

To assess the prevalence of strain ATCC 33150 in existing oral microbiome data sets, we developed a custom Kraken2 mini-database capable of resolving four *Selenomonas* strains (ATCC 33150, ATCC 35185, KCOM 20461, and KCOM 1787) at the subspecies level. Applying this database to reads previously classified as *S. sputigena* (TaxID 69823) in three independent cohorts, we found that ATCC 33150 was detectable in all samples across all data sets (Fig. 2).

To assess the robustness and consistency of the *Selenomonas* strain enrichment signal across independent data sets, a combined cohort analysis was performed using a linear mixed model (LMM). Healthy samples from the Saito et al. (*n* = 13) and Belstrøm et al. metagenome (*n* = 8) cohorts were pooled with periodontitis samples from all three data sets (Saito *n* = 14, Belstrøm *n* = 9, and Shi *n* = 24), yielding 21 healthy and 47 periodontitis samples across three data sets and two sample types (saliva and subgingival plaque) (35–38). The model log1p(RPM) ~ group + sample_type + (1|data set) was fitted for each strain separately, with group and sample_type as fixed effects and data set as a random intercept. All four *Selenomonas* strains and the species-level total were significantly elevated in periodontitis compared to healthy controls (LMM, all *P* ≤ 0.0008). For ATCC 33150, the estimated effect size was beta = 1.458 (*t* = 3.508, *P* = 0.0008), corresponding to a median reads per million (RPM) of 10.1 in healthy samples and 77.0 in periodontitis samples (~7.6-fold enrichment). Similar effect sizes were observed for ATCC 35185 (beta = 1.652, *P* = 0.0001), KCOM 20461 (beta = 1.791, *P* = 0.0001), KCOM 1787 (beta = 1.702, *P* = 0.0002), and the species-level total (beta = 1.749, *P* = 0.0001). *S. sputigena* strains ATCC 35185, KCOM 20461, and KCOM 1787 were enriched in the periodontitis samples (7.7×, 8.3×, and 8.0×, respectively). These findings demonstrate that the enrichment of all four *Selenomonas* strains in periodontitis is robust to differences in sample type (saliva vs subgingival plaque) and between-study variability, supporting a consistent and reproducible association between ATCC 33150 and periodontal disease across geographically and clinically diverse cohorts.

To provide a broader microbiological context for the *Selenomonas* findings, the abundance of 12 periodontal bacteria with detectable reads across all data sets was assessed in the Saito et al. and Belstrøm et al. metagenome cohorts, as well as in the Shi et al. subgingival plaque cohort (Fig. S1). Four organisms (*Capnocytophaga granulosa, Capnocytophaga haemolytica, Campylobacter rectus, and Eubacterium nodatum*) had zero reads in one or more groups and were excluded from statistical analyses. We observed significantly elevated abundance of *Capnocytophaga ochracea* (*P* = 9.5 × 10^−6^), *Fusobacterium nucleatum* (*P* = 3.1 × 10^−6^), *Treponema denticola* (*P* = 1.3 × 10^−5^), *Tannerella forsythia* (*P* = 2.6 × 10^−4^), *Prevotella intermedia* (*P* = 1.3 × 10^−4^), and *Porphyromonas gingivalis* (*P* = 0.0132). The detection of significant enrichment of periodontal pathogens in the same samples that show elevated *S. sputigena* and ATCC 33150 abundance supports the biological plausibility of ATCC 33150 as a potential periodontal disease-associated organism.

### The genomes of *S. sputigena* ATCC 35185 and the *Selenomonas* sp. ATCC 33150 harbor distinct prophage elements

We queried the whole-genome sequences of both ATCC 35185 and ATCC 33150 for prophage elements using PHASTEST (39). This analysis identified one contiguous prophage region for each genome, and each genome contained a different prophage. ATCC 35185 harbored a prophage sequence homologous to *Salmonella* phage SEN34 (RefSeq: NC_028699), located at nucleotide position 1,466,963–1,519,811 (Fig. S2). ATCC 33150 harbored a prophage sequence homologous to *Aeribacillus* phage AP45 (RefSeq: NC_048651), located at nucleotide position 1148951–1187867 (Fig. S2). Interestingly, the ATCC 33150 prophage element is inserted within an operon that otherwise encodes for CRISPR-associated machinery.

### Functional enrichment analysis between isolates of *Selenomonas* spp

Johnson et al. (21) reported that ATCC 33150 can ferment arabinose, whereas ATCC 35185 cannot. We sought to determine whether differences in their genomic content may explain this distinction and identify other significant differences between the two oral *Selenomonas* species. We conducted a functional enrichment analysis to identify clusters of orthologous genes present only in ATCC 33150 or unique to the *S. sputigena* strains (ATCC 35185, KCOM1787, and KCOM20461) (Table S1). We identified 62 COGs present in the ATCC 33150 genome but absent in *S. sputigena*. Similarly, we identified 41 COGs encoded in all *S. sputigena* genomes but absent from the ATCC 33150 genome.

Most striking is the increased saccharolytic potential of ATCC 33150 compared to *S. sputigena*. Consistent with the prior observation that ATCC 33150 uniquely ferments arabinose, ATCC 33150 encodes an L-arabinose isomerase (EC:5.3.1.4), the enzyme that catalyzes the first step in the L-arabinose utilization pathway (nucleotide location 739,621–741,046) (Table S1). This function is only encoded by ATCC 33150 and *S. flueggei* ATCC 43531 among oral *Selenomonas* spp. (Table S2). The ATCC 33150 genome also encodes a putative rhamnose utilization operon that includes a major facilitator superfamily transporter (nucleotide locations 66,197–71,566). Another extensive collection of COGs unique to ATCC 33150 is contained within an operon (nucleotide location 265,687–271,277) that encodes phosphotransferase system components for the transport of sorbitol and dulcitol, as well as a homolog of GutM. This major transcriptional activator regulates the transport and utilization of sorbitol in *Escherichia coli* and *Lactobacillus casei* (40, 41). Two additional clusters of unique COGs (nucleotide location 1,072,374–1,083,569 and 1,325,525–1,333,762) encode ribose transporters and enzymes, along with homologs of the YesMN two-component system that regulates carbohydrate utilization (42, 43).

The two species also differ in their capability to synthesize biotin (vitamin B7), an essential cofactor for many enzymes involved in central metabolism (44). *S. sputigena* ATCC 35185 encodes the complete biosynthetic pathway to produce biotin from pimelate. In contrast, ATCC 33150 is likely auxotrophic for biotin, as it lacks genes homologous to BioW, BioF, or BioA, suggesting it cannot utilize pimelate. ATCC 33150 likely acquires biotin from its environment, as it encodes BioY. This transporter can import either biotin or its precursor, dethiobiotin, and, together with BioB, which ATCC 33150 encodes, converts dethiobiotin into biotin. The ability to synthesize its own essential vitamin may provide a fitness advantage for *S. sputigena*.

The physiology of most bacteria depends on iron acquisition, as iron is an essential component of many enzymes, and heme-dependent cytochromes are critical to respiration. *S. sputigena* appears to possess a wider repertoire of iron acquisition systems than ATCC 33150. The *S. sputigena* ATCC 35185 genome encodes 12 annotated iron-complex outer membrane receptor proteins that transport siderophores across the outer membrane. The other *S. sputigena* strains (KCOM1787 and KCOM20461) also encode 8 and 10 iron complex receptors, respectively. Interestingly, ATCC 33150 only encodes two outer membrane iron receptors. Diderm bacteria must also transport iron across the inner membrane, which is often accomplished through specific ATP-binding cassette transporters consisting of at least one soluble substrate-binding protein (SBP). Again, all three *S*. *sputigena* strains encode seven to eight iron complex-specific SBPs, while ATCC 33150 only encodes four. Interestingly, ATCC 33150 uniquely encodes the PdtaSR two-component system, which senses intracellular iron levels in *Mycobacterium* spp., suggesting ATCC 33150 may sense and regulate iron metabolism differently from *S. sputigena* (45). The extent to which differences in genomic content devoted to iron acquisition influence the growth and fitness of these organisms in iron-depleted environments remains unknown.

Several other potential functions are absent from ATCC 33150 but present in *S. sputigena*. A cluster of unique COGs encoding the putative type IV secretion apparatus is only found in ATCC 35185 (nucleotide location 721,445–730,229). Notably, the putative type IV secretion system-associated genes are absent from the KCOM1787 and KCOM20461 strains (Table S2). Whether the type IV secretion system of *S. sputigena* ATCC 35185 is complete or how it functions remains unknown. The *S. sputigena* ATCC 35185 genome also encodes unique COGs associated with polysaccharide biosynthesis that are absent in ATCC 33150. One cluster (nucleotide location 32,191–36,783) encodes four genes in an operon in the ATCC 35185 genome associated with polysaccharide biosynthesis and export. Homologs of the polysaccharide biosynthesis proteins PelF and PelG were also identified in all *S. sputigena* strains but were absent in ATCC 33150.

### ATCC 33150 has reduced growth in TYGVS compared to *S. sputigena* ATCC 35185

**Fig 3:**
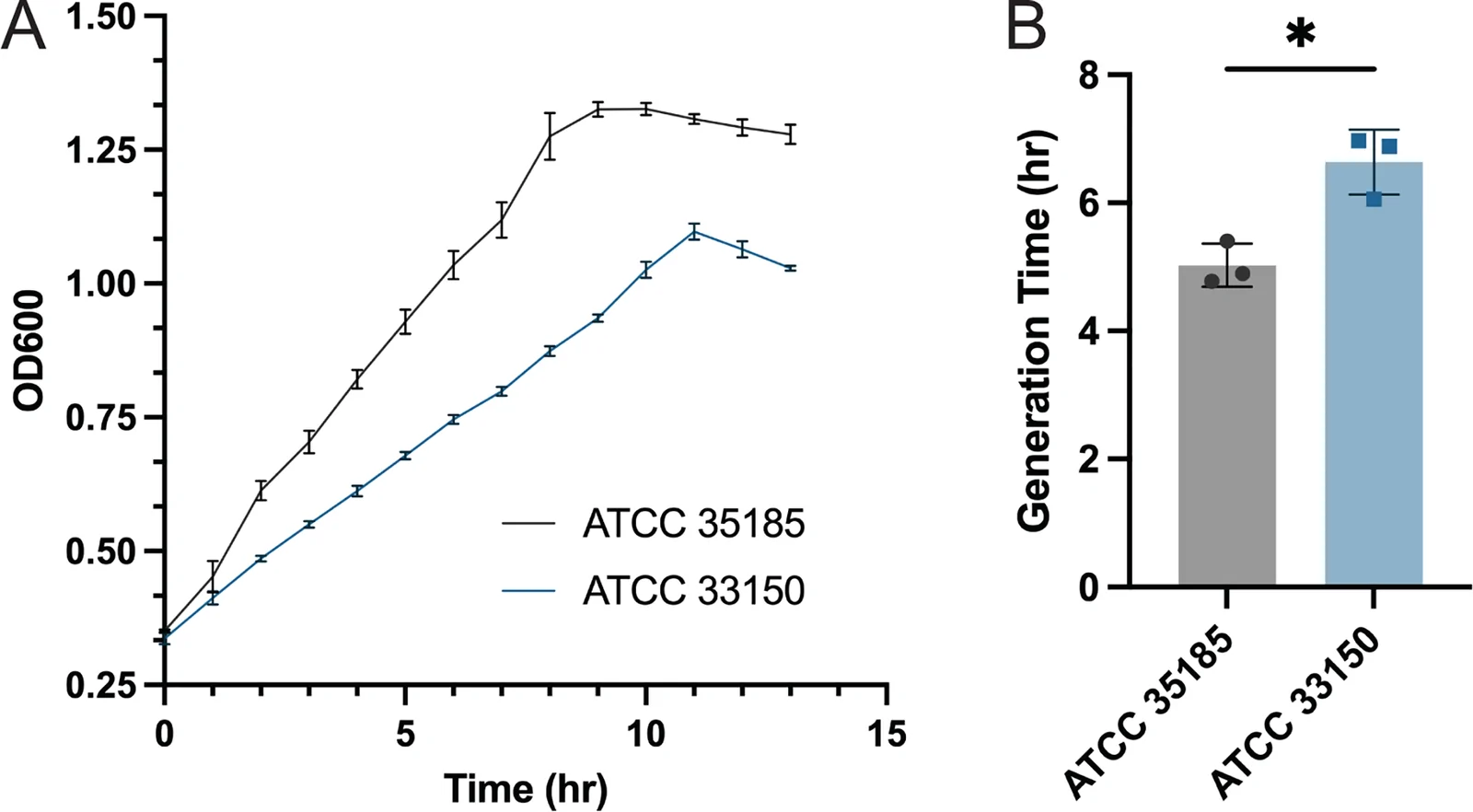
ATCC 33150 grows more slowly than *S. sputigena* ATCC 35185. (**A**) The linear growth of both strains was measured over 13 h in TYGVS broth, and (**B**) the generation time was determined. ATCC 33150 grew more slowly and reached a lower final density than *S. sputigena* ATCC 35185. The data are the average of three biological replicates with standard deviation. The generation time data were analyzed using a Student’s *t*-test (*P < 0.05).

Our genomic comparisons revealed differences in metabolic capabilities, biotin biosynthesis, and the potential to acquire iron, suggesting that the two selenomonads may exhibit different growth *in vitro*. To compare logarithmic growth between the two isolates, tryptone-yeast extract-gelatin-volatile fatty acids-serum (TYGVS) broth was inoculated with ATCC 35185 and ATCC 33150 at 3.5 × 10^8^ cells per mL and incubated anaerobically at 37°C for 13 h (Fig. 3). The *S. sputigena* ATCC 35185 cultures achieved a higher cell density than the ATCC 33150 cultures. *S. sputigena* had a shorter generation time than ATCC 33150 and ended logarithmic growth between 9 and 10 h, while ATCC 33150 remained in logarithmic growth for 12 h (Fig. 3). Direct cell counts determined the culture density of both strains to be 4.8 × 10^7^ cells per mL at 0.05 optical density (OD) at 600 nm (Fig. S2). Scaling for the dilution factor, both isolates have a culture density of 9.6 × 10^8^ cells per mL at 1.0 OD at 600 nm, very close to the widely accepted 1 × 10^9^ cells per mL for most spherical or rod-shaped bacteria.

### ATCC 33150 exhibits extensive autoaggregation but does not form robust surface-attached biofilms *in vitro*

**Fig 4:**
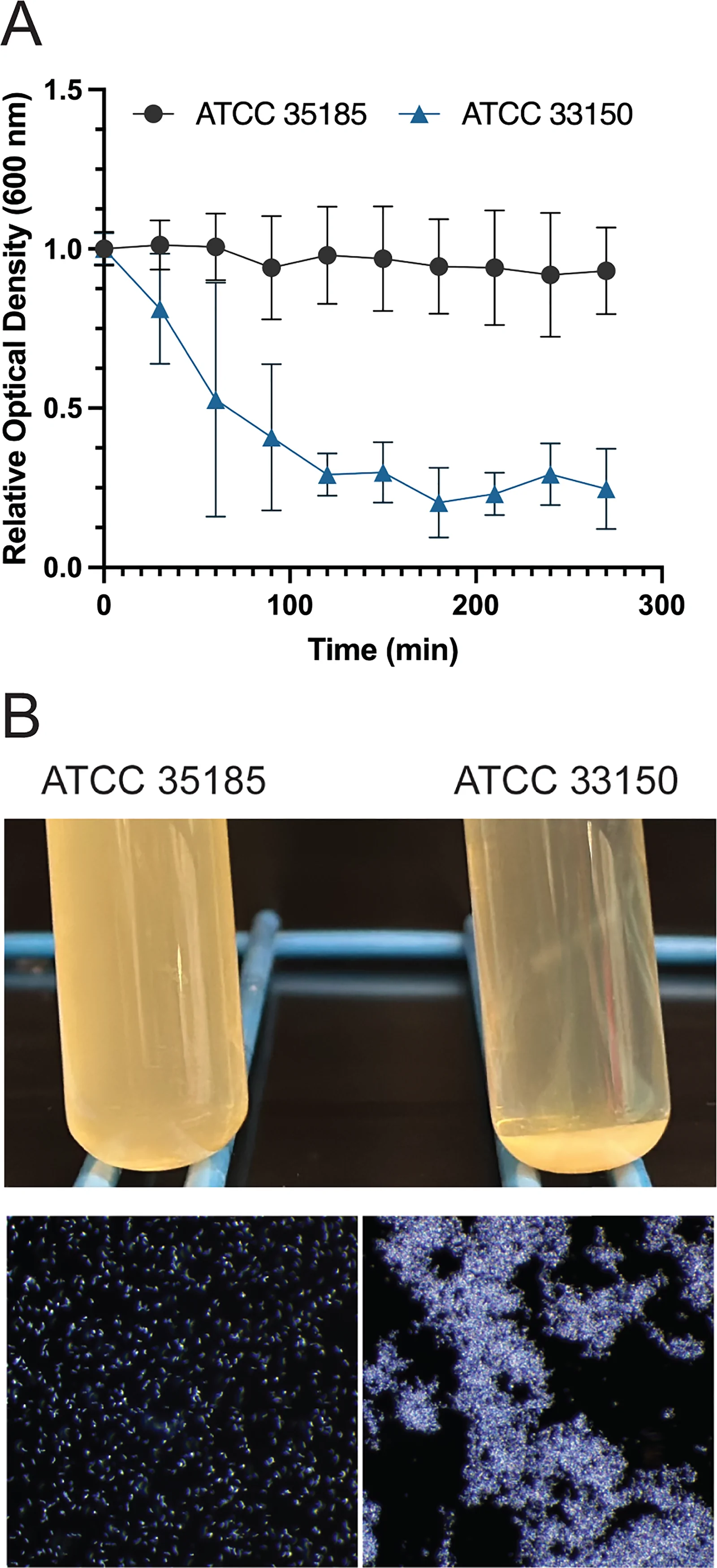
ATCC 33150 displays robust autoaggregation in TYGVS broth. (**A**) ATCC 35185 and ATCC 33150 were grown to late-log phase in TYGVS. The culture tubes were inverted to homogenize the cultures, and the top 500 μL of culture was removed every 30 minutes, and the optical density (600 nm) was recorded. The values were normalized to 0 minutes (immediately after homogenization) and are the average of three biological replicates with standard deviation. Data were analyzed using linear regression for both curves, followed by an *F*-test to determine whether the two models differed significantly (*P* = 0.0002). (**B**) The top panel shows a digital picture of the culture tube for each species after 170 minutes, and the bottom panel shows a dark-field image of the cells at the bottom of the culture tube using a 40× objective. Images were captured for each of the three independent experiments, and the images are representative of all experiments.

During routine cultivation, we observed that ATCC 33150 aggregates and flocculates out of suspension, settling at the bottom of the growth vessel, whereas *S. sputigena* ATCC 35185 remains suspended in solution (Fig. 4A). To better characterize this difference, we grew both species in TYGVS for 12 h (early stationary phase) before homogenizing the cultures and recording the optical density from the top of the tube every 30 minutes for 4.5 h (Fig. 4B). We observed that ATCC 33150 began aggregating and falling to the bottom of the tube within 30 minutes and had finished aggregating by 2 h, while the optical density at the top of the *S. sputigena* ATCC 35185 tube never significantly changed. We then visualized the aggregative properties of ATCC 33150 by collecting cells from the bottom of the tube after 4.5 h and examining them under dark-field microscopy. *S. sputigena* ATCC 35185 was nearly always visible as individual cells, while ATCC 33150 formed large clusters of aggregated cells (Fig. 4B).

**Fig 5:**
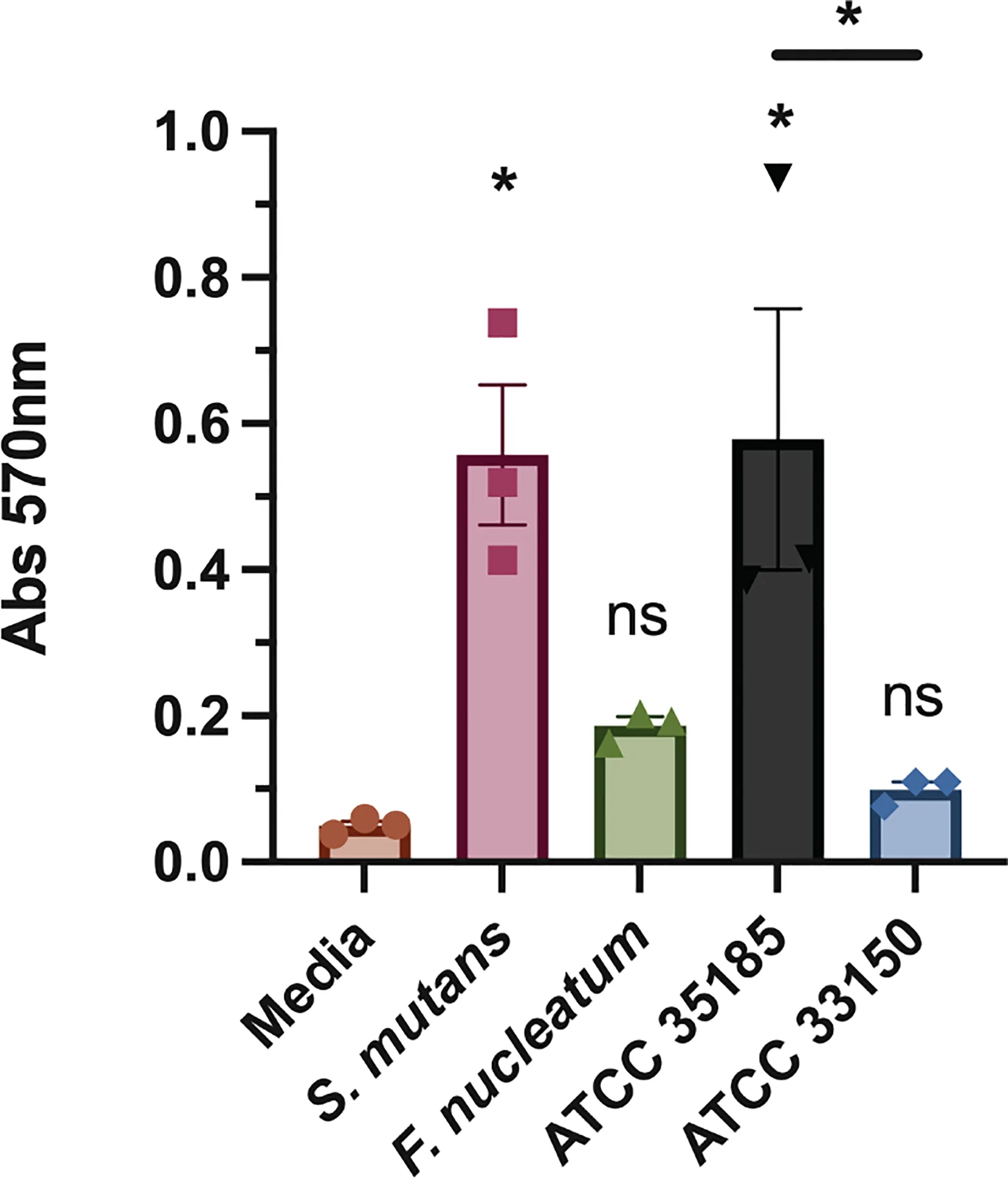
*S. sputigena* ATCC 35185 forms *in vitro* biofilms, while ATCC 33150 does not. *S. sputigena* ATCC 35185, ATCC 33150, *F. nucleatum* subsp. *nucleatum* ATCC 25585, and *S. mutans* UA159 were grown in 12-well plates for 18 h before the biofilms were washed, and the total biomass was stained with crystal violet. As a negative control, blank wells were incubated with medium alone. The stain was solubilized and quantified at 570 nm. The data represent the average of four biological replicates, along with the standard error of the mean. The data were analyzed by one-way ANOVA with Tukey’s *post hoc* test (*P < 0.05; ns, not significant). Labels above each bar are comparisons with the medium control.

*Selenomonas* spp. are present in both subgingival and supragingival plaque biofilms and are described as distributed throughout the biofilms and contributing to their architecture (33, 46, 47). Autoaggregation can be an early step in biofilm formation as planktonic cells coalesce into large aggregates, suggesting that ATCC 33150 may form more robust biofilms than *S. sputigena* ATCC 35185, owing to its greater aggregative nature. Conversely, the *S. sputigena* strains (ATCC 35185, KCOM1787, and KCOM20461) encode COGs associated with polysaccharide production (Table S1) that are absent in ATCC 33150. To determine whether the two *Selenomonas* spp. make monospecies biofilms, we developed an *in vitro* biofilm, stained it with crystal violet, and observed that *S. sputigena* ATCC 35185 formed robust surface-adherent biofilms, whereas ATCC 33150 did not (Fig. 5). *S. mutans* is a robust biofilm-producing species associated with dental caries (48, 49). *Fusobacterium nucleatum* is a key periodontitis-associated constituent of the subgingival biofilm, and the five subspecies of *F. nucleatum* have variable biofilm-forming capacity (50, 51). *F. nucleatum* subsp. *nucleatum* ATCC 25586, used in our study, is a low-moderate monospecies biofilm producer (50). Consistent with the literature, we observed robust biofilm production by *S. mutans* UA159 and less robust biofilm production by *F. nucleatum* ATCC 25586. The degree to which *S. sputigena* formed *in vitro* monospecies biofilms was comparable to *S. mutans*.

### ATCC 33150 is highly motile during *in vitro* cultivation

**Fig 6:**
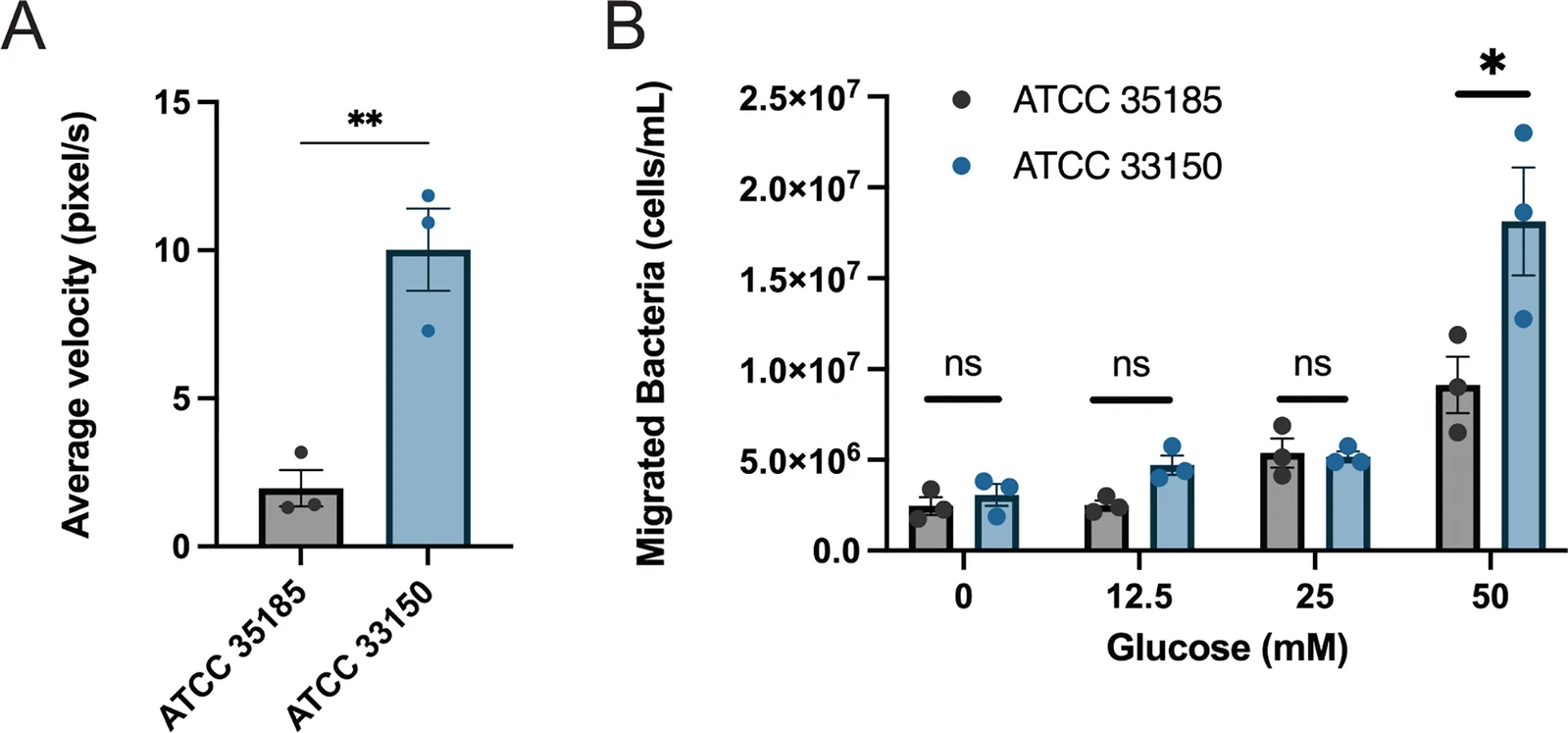
ATCC 33150 is more motile than *S. sputigena* ATCC 35185. (**A**) Cell tracking analysis of ATCC 33150 and *S. sputigena* ATCC 35185*. Selenomonas* spp*.* cells were tracked in TYGVS over 15 s. The data represent the average of cell velocities calculated from independent experiments (*n* = 3) with the standard error of the mean. Data were analyzed by *t*-test (**P < 0.01). (**B**) The number of cells per milliliter for ATCC 35185 or ATCC 33150 was assessed using a Petroff-Hausser chamber and dark-field microscopy after treatment with either 12.5, 25, or 50 mM glucose. Data are the average of three independent experiments with standard error of the mean and were analyzed by ANOVA with Tukey’s *post hoc* test (ns, not significant; *P < 0.05).

The genus *Selenomonas* is defined by crescent-shaped motile bacteria with a flagellar bundle localized to the concave surface of the cell. During routine culturing of ATCC 33150 and *S. sputigena* ATCC 35185, we observed pronounced differences in motility. Here, we quantified the swimming speed of ATCC 35185 and ATCC 33150 in TYGVS broth using dark-field microscopy. Motion-tracking software was used to quantitatively measure the movement of hundreds of bacterial cells from each species (Fig. 6A). Qualitatively, we seldom observed translational movement of *S. sputigena* ATCC 35185 in broth, whereas productive movement of ATCC 33150 was common. Quantitative measurement of velocity confirmed that ATCC 33150 swam 5.5 times faster than *S. sputigena* ATCC 35185. Representative movies for each species are shown in the Movies S1 and S2.

We observed that ATCC 33150 consistently exhibited productive motility, whereas *S. sputigena* ATCC 35185 was predominantly non-motile in TYGVS broth. The *S. sputigena* ATCC 35185 genome encodes several putative flagellin-like proteins (SELSP_0329, SELSP_0429, and SELSP_0433), each with an ortholog in the ATCC 33150 genome that shares 92.5%, 79%, and 83.6% identity, respectively. Functional enrichment did not identify any motility-associated genes unique to ATCC 33150 (Table S1). All three *S. sputigena* genomes encode a putative flagellar brake protein that is absent in ATCC 33150 (Table S2). However, it is untested whether this gene functions as a flagellar brake. Collectively, there is no obvious genomic explanation for the stark contrast in morphology phenotypes, and it likely represents an environmental or regulatory difference.

We then sought to determine if the difference in motility would impact chemotaxis in response to glucose. We observed that ATCC 33150 responded twofold more to 50 mM glucose than *S. sputigena* ATCC 35185 (Fig. 6B). Interestingly, glucose appeared to be only a weak chemoattractant, as neither species showed a strong response to glucose concentrations. For *S. sputigena* ATCC 35185, we observed a slight but statistically insignificant increase in the number of migrated bacteria between 25 and 50 mM glucose. Collectively, these results demonstrate that ATCC 33150 is more motile than *S. sputigena* ATCC 35185 under *in vitro* conditions.

### Both *Selenomonas* species can tolerate acidic environments

**Fig 7:**
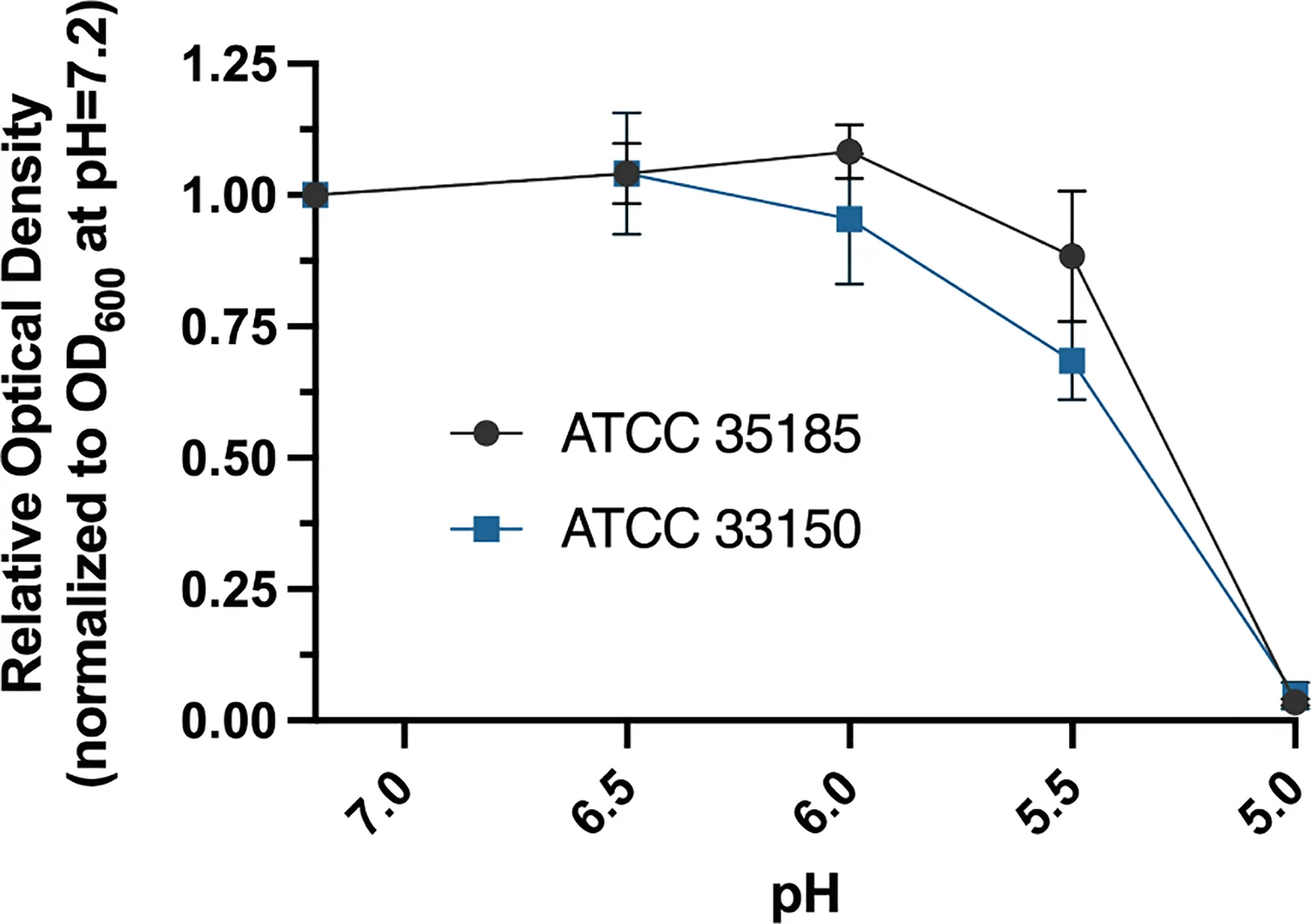
Both species display similar acid tolerance**.** pH-adjusted TYGVS broth was inoculated with an equal number of cells (1 × 10^8^) of both species. Cultures were grown anaerobically for 18 h, and growth was quantified spectrophotometrically (600 nm). All measurements were normalized to growth at pH 7.2 to account for the difference in growth rates between the two species. Data are the mean (*n* = 3) with the standard error of the mean and were analyzed by nonlinear regression, followed by an *F*-test (*P* = 0.0432).

Cho et al*.* (33) identified *S. sputigena* as a constituent of early-childhood supragingival biofilms, likely contributing to caries. In fact, they demonstrated that *S. sputigena* ATCC 35185 exacerbates *S. mutans*-dependent cariogenesis in a murine model. The same study described that *S. sputigena* becomes entrapped in the extracellular polysaccharide matrix produced by *S. mutans,* where *S. sputigena* proliferates and enhances the acidogenesis of *S. mutans*. Here, we sought to determine whether both *Selenomonas* species can tolerate the acidic conditions required for caries development (Fig. 7). Both ATCC 33150 and *S. sputigena* ATCC 35185 grew equally well at pH 7.2 and 6.5, and neither isolate grew at pH 5.0, consistent with the previous report that *S. sputigena* ATCC 35185, in pure culture, cannot grow below pH 5.5 (33). We observed slight differences in optical density between the two *Selenomonas* spp. at pH 6.0 and 5.5. Nonlinear regression analysis revealed a significant difference (*P* = 0.0432) between the two species, suggesting ATCC 33150 may be less aciduric than *S. sputigena* ATCC 35185.

## DISCUSSION

In this study, we initially sought to characterize ATCC 33150, presumed to be a strain of *Selenomonas sputigena*, to better understand how it compares to ATCC 35185 in virulence-associated phenotypes that may contribute to periodontitis. Average nucleotide identity analysis comparing the genome of ATCC 33150 to ATCC 35185 demonstrated that strain ATCC 33150 does not belong to the species *S. sputigena*. This is consistent with reports from more than 20 years ago suggesting that ATCC 33150 is not representative of *S. sputigena* and that it had never been exhaustively described. Once we established that ATCC 33150 is not *S. sputigena*, we sought to compare it with other named and cultivable *Selenomonas* spp*.* using ANI. We demonstrated that ATCC 33150 is a distinct new species belonging to the genus *Selenomonas* and propose the name *Selenomonas lamontii*.

The previous report by Johnson et al. (21) found that repeated attempts to cultivate another isolate with similarities to ATCC 33150 from 21 subgingival plaque samples failed and concluded that ATCC 33150 likely represented a rare oral species. We reanalyzed previously published metagenomic data sets and identified *S. lamontii* ATCC 33150 reads in all samples, demonstrating that *S. lamontii* is likely a common constituent of the human oral microbiome. We fully acknowledge the limitations of combining data sets produced under different experimental conditions and were cautious in interpreting these results. We can confidently state that ATCC 33150 is not rare and represents a common member of the human oral microbiome. Interestingly, most saliva samples mapped to multiple *Selenomonas* genomes, consistent with a recent report of multiple *P. gingivalis* strains within samples (52).

Our analysis of the combined data set revealed significantly higher abundance of both *S. lamontii* and *S. sputigena* in samples collected from periodontitis patients. This is in strong agreement with a recent review and reanalysis of hundreds of clinical samples, which found that *Selenomonas* abundance is elevated in gingivitis and periodontitis compared with healthy individuals (53). Again, care should be taken in interpreting these data. The importance of this study is to highlight that ATCC 33150 represents a novel species, *S. lamontii*, that may be a common member of the oral microbiome. Our data suggest that its abundance may increase during periodontitis. Future metagenomic, metatranscriptomic, and murine periodontitis model studies will be needed to determine whether *S. lamontii* is a contributor to periodontitis or an inflammophilic passenger.

Our results indicate a stark difference in the tendency of *S. sputigena* ATCC 35185 and *S. lamontii* ATCC 33150 toward a sessile or motile lifestyle *in vitro*. We observed that *S. lamontii* ATCC 33150 motility is more pronounced than that of *S. sputigena* ATCC 35185, which correlates with the more robust biofilm development exhibited by *S. sputigena* ATCC 35185. The secondary messenger bis-(3′−5′)-cyclic diguanylate (c-di-GMP) is a master regulator in lifestyle switching. In general, intracellular c-di-GMP levels act as a rheostat, promoting motility at low concentrations and inhibiting motility at higher concentrations, while promoting biofilm development (54). Diguanylate cyclase (DGC) enzymes, containing the GGDEF domain, synthesize c-di-GMP, while phosphodiesterases (PDE) hydrolyze it (55). To exert its regulatory effects on bacterial physiology, c-di-GMP binds to protein receptors and riboswitches, thereby modulating cellular processes and gene expression (56). *S. sputigena* and *S. noxia* produce c-di-GMP; however, this signaling mechanism remains unstudied in *Selenomonas* spp. (57). Comparing the annotated protein functions of *S. lamontii* ATCC 33150 to *S. sputigena* ATCC 35185, we did not find significant differences in putative c-di-GMP signaling. Both genomes contained genes annotated as either a DGC or containing the GGDEF, and both genomes contained a single gene annotated as a PDE. The genome annotations do not indicate a discernible difference in c-di-GMP signaling between the two *Selenomonas* species, and it is important to note that none of these protein functions have been experimentally tested. This remains a critical signaling system; future studies should investigate the nature of c-di-GMP regulation in *Selenomonas* spp.

We recently described the attachment and internalization of *S. sputigena* ATCC 35185 when the bacteria are incubated with gingival keratinocytes. In this study, we utilized the ATCC 33150 strain and observed a twofold difference in the internalization of *S. sputigena* ATCC 35185 and *S. lamontii* ATCC 33150 by challenged keratinocytes (31). It is likely that the significant difference in motility between these species results in variable attachment rates and subsequent internalization. The interaction of *Treponema denticola*, another periodontitis-associated bacterium, with host cells is increased in non-motile strains (58). Considering how little we know about the physiology and surface adhesins of either species, the differences observed in our previous study may be multifactorial. Future research into *Selenomonas* spp*.* will provide important insights into the biology of these unique bacteria.

Regarding motility, it was striking that neither *S. sputigena* ATCC 35185 nor *S. lamontii* ATCC 33150 demonstrated significant chemotaxis toward glucose concentrations less than 50 mM. For comparison, we recently observed significant chemotaxis of the oral spirochete *T. denticola* in response to 0.1 mM glucose (59). *E. coli* was also reported to display robust chemotaxis in response to micromolar concentrations of glucose (60). An important consideration for this study is that glucose suppresses flagellation of *Selenomonas ruminantium* (61). Although the effect on motility was not assessed in *S. ruminantium*, catabolite repression of motility may be a common trait among selenomonads. We did not examine whether glucose affects flagellar production in *S. sputigena* ATCC 35185 or *S. lamontii* ATCC 33150. Alternatively, glucose may not be a chemoattractant for either species. No study has mechanistically examined the motility or chemotaxis of oral *Selenomonas* spp*.* Genomic analysis for both *S. lamontii* ATCC 33150 and *S. sputigena* ATCC 35185 revealed that both species possess a conserved region adjacent to the flagellar biosynthetic operon that possesses homologs of CheA, CheY, CheW, CheC, and CheD, and both genomes encode numerous proteins predicted to be methyl-accepting chemotaxis proteins (Table S2). Many components required for chemotaxis are present in both genomes, suggesting a more nuanced explanation for our observation that *Selenomonas* spp*.* chemotaxis to glucose requires 100-fold more chemoattractant than *T. denticola*. Future studies are needed to better understand the unique motility of *Selenomonas*, a likely virulence-associated phenotype.

It is essential to consider that periodontitis is not only a result of an increased bacterial burden or dysbiosis but also synergistic interactions among microbiome constituents that increase the pathogenic potential of the community beyond the sum of its parts (62–64). Drescher et al. (46) described *Selenomonas* as present throughout the subgingival biofilm and contributing to the biofilm architecture. Our results suggest that *S. sputigena* may be more likely to contribute to polymicrobial biofilms than *S. lamontii. Selenomonas* spp. are understudied, and their interactions within a polymicrobial context are largely unknown. *S. sputigena*, *S. infelix*, *S. noxia*, and *S. flueggei* coaggregate with several strains *of Fusobacterium nucleatum* (47). However, the molecular mechanism behind this interaction has never been investigated. Many bacterial-bacterial interactions involving *F. nucleatum* involved lectin-like adhesins, and these interactions most commonly involve galactose or, in some cases, rhamnose (65). Interestingly, the *S. sputigena* flagella are heavily glycosylated with rhamnose-linked *O*-glycans, providing a potential interaction between the *S. sputigena* flagella and the *F. nucleatum* adhesins (66). Alternatively, as discussed below, lipid A of *Selenomonas* spp*.* uncommonly contains galactose, which may contribute to adherence to *F. nucleatum* (67). *S. sputigena* ATCC 35185 was recently demonstrated to interact with *S. mutans*. The presence of *S. sputigena* stimulated acid production in *S. mutans*, which resulted in exacerbated *S. mutans*-mediated cariogenesis in a mouse model (33). Our results demonstrate that *S. sputigena* and *S. lamontii* are similarly resistant to acidic growth conditions, suggesting *S. lamontii* could withstand the *S. mutans*-modified environment and contribute to cariogenesis. Again, future studies should investigate the diversity of interactions between *S. mutans* and various *Selenomonas* spp., including *S. lamontii*. It will be important in future studies to elucidate the mechanisms underlying *Selenomonas* interactions with *F. nucleatum* and other oral bacteria.

Lipopolysaccharides (LPSs) from gram-negative bacteria are major components of the outer membrane and act as potent activators of inflammatory responses. In 1986, Kurimoto et al. (68) investigated the chemical composition of LPS from *S. sputigena* isolated from human oral samples and compared it with that of *E. coli*. The chemical composition of *S. sputigena* LPS was found to be fairly typical; however, it was 100-fold more pyrogenic than *E. coli* LPS when injected into rabbits, and *E. coli* LPS required a 200-fold higher dose to provoke a positive Shwartzman reaction in rabbits (68). This study reported that their isolated strains fermented glucose, fructose, mannose, and maltose but did not produce acids from other carbohydrates, suggesting that these isolates were likely *S. sputigena*-like isolates. In 1997, Kumada et al. (67) chemically characterized the LPS from ATCC 33150. They described the lipid A moiety as containing significant amounts of galactosamine, in addition to the common glucosamine, suggesting that the lipid A of ATCC 33150 may have a backbone distinct from the typical β (1-6) glucosamine disaccharide. Kurimoto et al. (68) only reported LPS composition as “16.5% hexosamine,” suggesting that they may not have characterized the LPS with enough depth to determine whether the atypical lipid A backbone described for *S. lamontii* ATCC 33150 is unique to this isolate or is more general among *Selenomonas* spp. (67). Considering LPS is likely a significant virulence factor promoting periodontal inflammation and likely contributed to fatal cases of *Selenomonas*-associated septicemia (26–28), future studies should seek to better characterize the LPS and lipid A composition from different *Selenomonas* spp*.*

The availability of the ATCC 33150 genome and ongoing efforts to develop a transformation protocol with *Selenomonas* spp*.* will make genetic manipulation of both *S. sputigena* and *S. lamontii* an immediate goal. This will enable future research to determine if some of the unique genes identified in this study contribute to the observed phenotypes, such as biofilm formation and motility. The availability of high-quality genomic sequence data, novel methodological tools, and unique insights into the behaviors of these organisms will collectively lead to future studies that can assess aspects of *Selenomonas* physiology, their unique motility, and virulence properties, all of which can cause disease above and below the gum line.

## MATERIALS AND METHODS

### Bacterial strains and growth conditions

The two *Selenomonas* strains used for *in vitro* experiments in this study are ATCC 35185 and ATCC 33150; both strains were purchased from the American Type Culture Collection. *Selenomonas* strains were cultured in tryptone-yeast extract-gelatin-volatile fatty acids-serum growth medium (32). *Streptococcus mutans* UA159 was generously provided by Dr. Todd Kitten, VCU Philips Institute for Oral Health Research. *Fusobacterium nucleatum* subsp. *nucleatum* ATCC 25586 was generously provided by Dr. Janina Lewis, VCU Philips Institute for Oral Health Research. *S. mutans* and *F. nucleatum* were cultured in supplemented brain heart infusion medium (33). Bacteria were grown anaerobically at 37°C to the mid-log phase before use in the experiments described. Bacterial culture purity was validated microscopically, and cultures were passaged no more than four times before re-establishing a new culture from a frozen stock.

### Logarithmic growth and aciduricity analysis

To determine cell density relative to optical density for the two isolates, bacteria were grown to the mid-log phase and then adjusted to an equal optical density of 0.05 at 600 nm. Direct cell counts were determined using a Petroff-Hauser counting chamber and dark-field microscopy. Each strain was counted in triplicate, and the data were analyzed using a *t*-test. For growth analysis, bacteria were cultured to the mid-log phase and then adjusted to an initial OD of 0.30 at 600 nm. The adjusted cultures were subsequently grown anaerobically at 37°C. The optical density of the cultures was recorded at 1-hour intervals until each culture reached the stationary growth phase, at which point the optical density did not increase over the course of three consecutive readings. For aciduricity analysis, bacteria were cultured to the mid-log phase, and afterward, 1 × 10^8^ bacteria were inoculated in 10 mL of pH-adjusted TYGVS medium. The pH range of pH-adjusted TYGVS used is as follows: 7.2, 6.5, 6.0, 5.5, and 5.0. The pH-adjusted cultures were grown overnight, and the subsequent growth was evaluated spectrophotometrically. To account for differences in growth rate between strains, the recorded optical densities were normalized to the OD_600_ of bacteria grown at pH 7.2. Both the growth curve and aciduricity analyses were repeated in three biological replicates. The generation time for ATCC 35185 and ATCC 33150 was analyzed using a *t*-test. Aciduricity was analyzed by nonlinear regression, and the curves were compared with an *F*-test.

### Analysis of motility and chemotaxis

For motility comparisons, bacteria were grown to the mid-log phase, and a dilution of approximately 1 × 10^6^ bacteria in 10 μL phosphate-buffered saline (PBS) was prepared. The diluted bacteria were promptly visualized via dark-field microscopy (Olympus model BX53, Olympus dry dark field condenser model U-DCD, 40× objective), and a 15-second movie was recorded using an Olympus DP74 camera. Movies were prepared for three separate biological replicate bacterial cultures for each *Selenomonas* species. The resulting movies were analyzed to determine the average velocity of the imaged bacteria using the CellSens Dimension motion tracking image analysis software (Evident Scientific). Default CellSens Dimension detection parameters were used for the “Detect objects” and “Start tracking” functions, and “Mean (Track Velocity)” was recorded for each bacterium identified. To account for the false-positive detection of non-motile bacteria, which move via capillary flow in solution, the average velocity of capillary flow-traveling bacteria was computed. The computed average capillary velocity was subtracted from the total velocity of each bacterium, such that non-motile capillary-traveling bacteria display a net velocity equal to approximately zero, and genuine motile bacteria display a net velocity greater than zero. For chemotaxis comparisons, the capillary assay for chemotaxis was employed as described previously (69). Glucose chemoattractant solutions were prepared in PBS, with glucose concentrations of 50, 25, 12.5, and 0 mM. Chemoattractant solutions were pre-reduced overnight prior to each experiment. Microhematocrit capillary tubes (Globe Scientific) were filled with 200 μL of pre-reduced chemoattractant and subsequently sealed with Cha-seal capillary tube sealant (Fisher Scientific). Bacteria were cultured to the mid-log phase and subsequently diluted in pre-reduced PBS to a final concentration of 5 × 10^6^ bacteria per well, with a final volume of 100 μL per well in a 96-well plate. Chemoattractant-prepared capillary tubes were inserted into each well, and the plate was incubated anaerobically at 37°C for 2 h. Afterward, the contents of each capillary tube were expelled, and 2 μL of solution from each capillary tube was loaded onto a Petroff Hauser counting chamber (Electron Microscopy Sciences) for enumeration via dark-field microscopy. The chemotaxis experiment described was repeated in three separate biological replicate experiments.

### Observing and quantifying autoaggregation of ATCC 33150

*Selenomonas* sp. ATCC 33150 and *S. sputigena* ATCC 35185 were grown in TYGVS broth under anaerobic conditions to mid-log phase. The culture tubes were tightly sealed and gently inverted 10 times to disperse any aggregates clumped at the bottom. Every 30 minutes, 500 μL of culture was removed from the top of the tube, and the absorbance was measured (600 nm) for a total of 4.5 h. After the 4.5-hour incubation, representative pictures of the culture tubes were taken with a digital camera, and representative images of the bacterial cells at the bottom of the culture tubes were captured using dark-field microscopy with an Olympus BX53 microscope equipped with a 40× objective and an Olympus DP74 digital camera. Initial absorbance values were normalized to 1 to enable direct comparisons, accounting for differences in growth rates and culture densities between the two *Selenomonas* spp. The data are the average of three independent experiments with a standard deviation. The data were analyzed by performing linear regression on each curve and comparing the two curves using an *F*-test (GraphPad Prism 11.0.0). Digital and dark-field images were captured for each experiment, and the images presented are representative of each experimental replicate.

### Analysis of biofilm and community formation

The crystal violet assay, used for detecting community formation, was employed to assess differences in community and biofilm formation as described previously (70). As positive controls for exopolysaccharide deposition and community formation, respectively, *Streptococcus mutans* and *Fusobacterium nucleatum* ssp. *nucleatum* were included (50, 71). Briefly, all bacterial strains were grown to the mid-log phase, as described above, then adjusted to an optical density at 600 nm of 0.20, and 1 mL of each culture was plated in each well of a 12-well tissue culture-treated plate. Each of the four organisms tested was plated on a single 12-well plate, with each organism occupying three distinct technical replicate wells. Afterward, the resulting plates were parafilm-wrapped and cultured anaerobically at 37°C for 18 h. The plates were then processed using the crystal violet detection assay as described previously, and the absorbance at 570 nm was recorded using a microplate reader. The crystal violet assay described was repeated in four separate biological replicate experiments.

### Whole-genome sequence analysis

The complete genomes for *S. sputigena* ATCC 35185 (NCBI genome assembly ASM20840v1), *S. sputigena* KCOM1787 (ASM2601596v1), *S. sputigena* KCOM20461 (ASM2601384v1), *Selenomonas* sp. ATCC 33150 (ASM516034v1), *S. artemidis* DSM19719 (ASM42666v1), *S. dianae* ATCC 43527 (ASM3952160v1), *S. flueggei* ATCC 43531 (ASM16069v1), *S. infelix* ATCC 43532 (Sele_infe_ATCC_43532_V1), *S. noxia* ATCC 43541 (ASM16055v1), *S. timonae* Marseille-Q3039 (ASM1425047v1), and *S. ruminantium* TAM6421 (ASM4240v1) were downloaded from NCBI and imported using previously described methods in the Anvi’o workflow (72). Functional enrichment analysis and ANI calculations were performed using the pyANI function within the Anvi’o workflow (73). The resulting ANI calculations were used to construct a phylogenetic tree using the Interactive Tree of Life phylogenetic tree display tool (74). Metabolic pathway completeness was estimated using the anvi-estimate-metabolism function within Anvi’o (75). Prophage detection was accomplished via the PHASTEST prophage detection tool (39).

### Metagenomics analysis—data sets

Three publicly available oral microbiome data sets were included in this analysis. The Saito et al. data set (BioProject PRJNA717815) comprised 27 metagenomic samples of nonstimulated saliva from participants in Brazil, of which 1 sample (SRR14122750) yielded zero sequencing reads and was excluded, leaving 13 healthy controls and 14 periodontitis cases for analysis (36, 37). The Belstrøm et al. data set (BioProject PRJNA396840) comprised 28 metagenomic samples of stimulated saliva from participants in Denmark, including healthy, caries, and periodontitis groups (35). Four samples (SRR5892214, SRR5892215, SRR5892201, and SRR5892237) were excluded due to corrupted Sequence Read Archive (SRA) files. The final metagenome cohort comprised eight healthy, nine caries, and nine periodontitis samples. The Shi et al. data set (BioProject PRJNA255922) comprised 48 subgingival dental plaque samples collected from 12 periodontitis subjects in the USA (38). Two affected tooth sites per subject were sampled at baseline (active periodontitis, Visit 1) and again after completion of scaling and root planning (SRP) therapy at a second clinic visit (resolved sites, Visit 2). Samples from the SRR1027xxxx accession series within the same BioProject were excluded due to unavailable metadata. This data set does not include naive healthy controls; all comparisons are therefore between active periodontitis and post-treatment resolved sites within the same subjects. Raw sequencing reads for both data sets were downloaded from the NCBI SRA using prefetch and fasterq-dump (SRA Toolkit).

### Metagenome analysis—taxonomic classification

Raw paired-end reads were classified using Kraken2 (v2.1.3) against the standard Kraken2 database (k2_standard_20260226), which includes complete bacterial, archaeal, viral, and human reference genomes from NCBI RefSeq. Classification was performed as a SLURM array job on a high-performance computing cluster, generating per-sample report files and read-level output files. Species-level abundance re-estimation for classical periodontal pathogens was performed using Bracken (v2.9), with a read length parameter of 150 bp (-r 150), producing per-sample Bracken species reports. Total reads per sample were calculated as the sum of classified and unclassified reads from the Kraken2 reports and used for normalization.

### Metagenome analysis—strain-level analysis

Standard Kraken2 classification cannot distinguish *Selenomonas* strains at the subspecies level because multiple strains, including ATCC 33150 (NZ_CP194411.1), share the same NCBI species-level taxonomy ID (TaxID 69823). To resolve individual strains, a custom two-pass classification strategy was developed.

#### Custom mini database construction

A strain-resolved Kraken2 mini database was constructed using four *Selenomonas* genome assemblies: ATCC 33150 (NZ_CP194411.1), ATCC 35185 (NC_015437.1), KCOM 20461 (NZ_CP110231.1), and KCOM 1787 (NZ_CP110383.1). Since ATCC 33150, KCOM 20461, and KCOM 1787 lack strain-level taxonomy IDs in NCBI, custom taxids (2000000001, 2000000002, and 2000000003, respectively) were assigned and appended to the nodes.dmp and names files.dmp taxonomy files copied from the standard database. FASTA headers for all four genomes were reformatted to the required Kraken2 format (>kraken:taxid|TAXID|ACCESSION) prior to library addition. The mini database was built using kraken2-build with the --build flag. Database validity was confirmed by classifying the ATCC 33150 genome against the mini database, which resulted in 100% of the sequence being assigned to the correct strain-level taxid (2000000001).

#### Two-pass classification

To avoid rebuilding the full standard database, a two-pass approach was employed. In the first pass, reads assigned to TaxID 69823 (*S. sputigena* species level) were extracted from the original Kraken2 read-level output files using awk. The corresponding paired-end reads were then retrieved from the original compressed FASTQ files using seqkit (v2.8.2) grep. In the second pass, these extracted reads were re-classified using Kraken2 against the custom mini-database, generating per-sample strain-level reports. This approach resolves each read to one of the four strains (or to the unresolved species level if no strain-specific assignment could be made) without reprocessing the full data set.

### Metagenome analysis—other oral bacteria analysis

The relative abundance of 16 periodontal bacteria was assessed using the per-sample Bracken species reports. Organisms were selected to provide a variety of established periodontal pathogens and other species associated with gingivitis or health (53). The analyzed species were *Porphyromonas gingivalis*, *Tannerella forsythia*, *Treponema denticola*, *Fusobacterium nucleatum*, *Prevotella intermedia*, *Parvimonas micra*, *Prevotella nigrescens*, *Capnocytophaga gingivalis*, *Capnocytophaga ochracea*, *Capnocytophaga sputigena*, *Eikenella corrodens*, *Aggregatibacter actinomycetemcomitans*, *Eubacterium nodatum*, *Capnocytophaga granulosa*, and *Capnocytophaga haemolytica*. Read counts for each organism were extracted from Bracken species reports by matching NCBI taxonomy IDs using awk. Organisms absent from all samples (zero reads across the entire data set) were excluded from downstream analyses.

### Metagenome analysis**—**normalization

All read counts were normalized to RPM by dividing the number of reads assigned to each taxon by the total reads per sample (classified plus unclassified) and multiplying by 10^6^. This normalization accounts for differences in sequencing depth between samples. Relative abundance was additionally expressed as a percentage of total reads (RPM/10,000) and as a percentage of total *Selenomonas* reads for strain-level comparisons.

### Metagenome analysis—statistical analysis

All statistical analyses were performed in R (v4.4.1) using the rstatix (v0.7.3) and ggplot2 (v4.0.0) packages. Differences in RPM between groups were assessed using the Wilcoxon rank-sum test, which was selected due to the non-normal distribution of metagenomic abundance data and the unequal group sizes. For two-group comparisons (healthy vs periodontitis), raw *P*-values are reported. Statistical significance was defined as *P* < 0.05. Fold change was calculated as the ratio of median RPM in the disease group to median RPM in the healthy/control group.

To assess the consistency of *Selenomonas* strain enrichment across independent cohorts and sample types, a combined cohort analysis was performed using a linear mixed model. Healthy and periodontitis samples from the Saito et al. metagenome cohort (*n* = 13 healthy, *n* = 14 periodontitis) and the Belstrøm et al. metagenome cohort (*n* = 8 healthy, *n* = 9 periodontitis) were combined with the periodontitis baseline samples from the Shi et al. cohort (*n* = 24 periodontitis; no healthy controls available), yielding a total of 21 healthy and 47 periodontitis samples. The Shi SRP samples were excluded from this analysis. RPM values were log-transformed using log1p prior to modeling. The LMM was specified as: log1p(RPM) ~ group + sample_type + (1|data set), where group (Health vs Periodontitis) and sample_type (Saliva vs Subgingival plaque) were included as fixed effects, and data set was included as a random effect to account for between-study variability. Models were fitted using the lme4 package, and *P*-values for fixed effects were obtained using the lmerTest package with Satterthwaite’s approximation for degrees of freedom.

## SUPPLEMENTAL MATERIAL

Supplemental figures spectrum.00341-26-s0001.docx Figures S1 to S3.

Table S1 spectrum.00341-26-s0002.xlsx The unique COGs identified for each isolate.

Table S2 spectrum.00341-26-s0003.xlsx The frequency of identified COGs for each species.

Movie S1 spectrum.00341-26-s0004.mp4 A representative movie of *S. sputigena* ATCC 35185 motility under darkfield microscopy. Bacteria were grown to mid-log phase, and a dilution of approximately 10^6^ bacteria in PBS was prepared. The diluted bacteria were promptly visualized by darkfield microscopy (Olympus BX53 microscope with a dry dark-field condenser model U-DCD, a 40× objective, and captured using the Olympus DP74 camera).

Movie S2 spectrum.00341-26-s0005.mp4 A representative movie of *S. lamontii* ATCC 33150 motility under darkfield microscopy. Bacteria were grown to mid-log phase, and a dilution of approximately 10^6^ bacteria in PBS was prepared. The diluted bacteria were promptly visualized by darkfield microscopy (Olympus BX53 microscope with a dry dark-field condenser model U-DCD, a 40× objective, and captured using the Olympus DP74 camera).

ASM does not own the copyrights to Supplemental Material that may be linked to, or accessed through, an article. The authors have granted ASM a non-exclusive, world-wide license to publish the Supplemental Material files. Please contact the corresponding author directly for reuse.
