# Supplementary material for: Genomic and phenotypic insights into the novel species Selenomonas lamontii type strain ATCC 33150, currently described as Selenomonas sputigena
Source: Microbiol Spectr. 2026 Jun 16;14(7):e00341-26. doi: 10.1128/spectrum.00341-26 (PMC13339959; doi:10.1128/spectrum.00341-26)
Supplement: Supplemental figures — Figures S1 to S3. [file spectrum.00341-26-s0001.docx]

**Supplemental Figures**

**
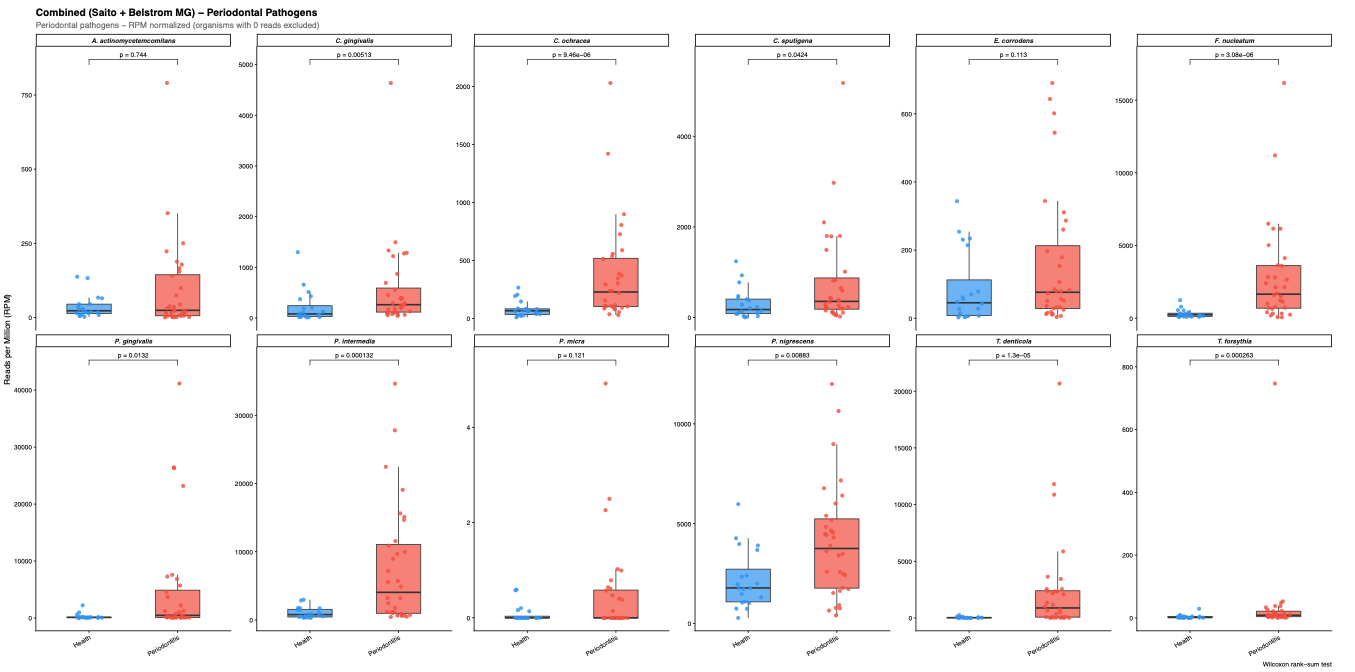
**

**Supplemental Figure 1. Abundance of periodontal bacteria in the LMM combined metagenomic dataset.** The abundance of 16 periodontal bacteria was determined using per-sample Bracken species reports. Organisms were selected to vary in their association with periodontal health or disease. Read counts were extracted using awk to match NCBI taxonomy IDs. All read counts were normalized to reads per million (RPM), models were fitted using the lme4 package, and p-values for fixed effects were obtained using the lmerTest package with Satterthwaite’s approximation for degrees of freedom.

**
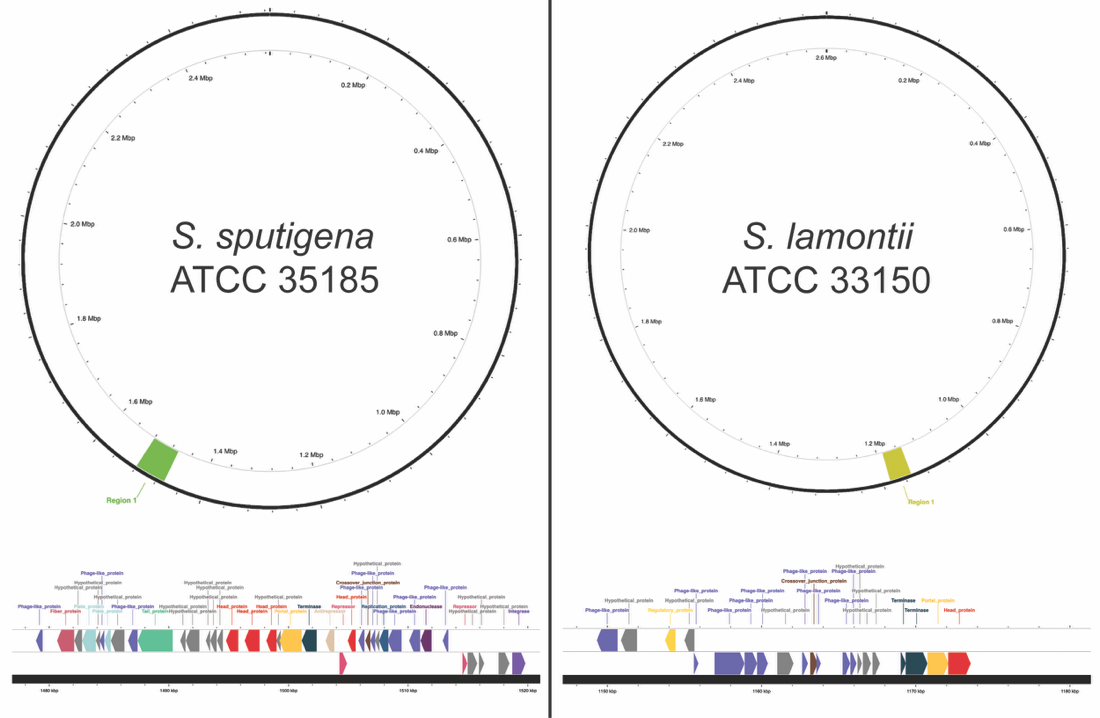
**

**Supplemental Figure 2. Both *Selenomonas spp.* genomes harbor complete but distinct prophages.** PHASTEST graphical viewer output for *S. sputigena* and *S. lamontii*. Whole genome sequences are represented as closed circular genomes. The colored boxes highlight the region of prophage insertion. *S. sputigena* prophage element is located at nucleotide position: 1466963-1519811, and is most similar to the *Salmonella* phage SEN34 (RefSeq: NC_028699). The *S. lamontii* prophage element is located at nucleotide position: 1148951-1187867, and is most similar to the *Aeribacillus* phage AP45 (RefSeq: NC_048651).


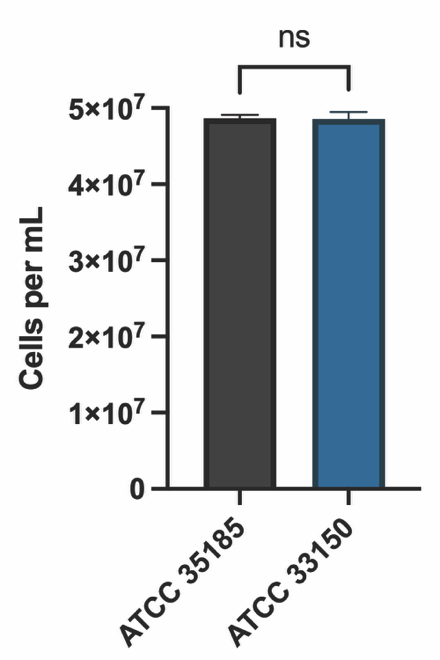


**Supplemental Figure 3. Cell density quantification from optical density-normalized cultures.** To determine whether optical density (OD) can be used to normalize cultures, mid-log-phase cultures were diluted to 0.05 OD and quantified by dark-field microscopy using a Petroff-Hauser counting chamber. The density in cells per mL for ATCC 35185 and ATCC 33150 was determined from triplicate cultures and compared using a t-test (ns: not significant).
